# The adaptation of academics to the Covid-19 crisis in terms of work time allocation

**DOI:** 10.1371/journal.pone.0273246

**Published:** 2022-08-24

**Authors:** Hugo Horta, Anna Panova, João Santos, Maria Yudkevich

**Affiliations:** 1 Social Contexts and Policies of Education, Faculty of Education, The University of Hong Kong, Hong Kong SAR, China; 2 Center for Institutional Studies, HSE University, Moscow, Russia; 3 Centro de Investigação e Estudos de Sociologia (CIES-Iscte), Iscte—Instituto Universitário de Lisboa, Lisbon, Portugal; Wuhan University, CHINA

## Abstract

Academics have seen their work environment and routines severely affected by the Covid19 pandemic. This topic has been analyzed by the literature, mostly from personal and descriptive perspectives, that highlight the challenging transitions and adaptations that academics have endured concerning their work and life-balance. This research complements those studies, by using a sample of university academics working all around the world in all disciplinary fields and focuses on a longitudinal perspective of workload and task time allocation of academic work. The findings show that academics which in general had long working hours, further increased their time of the week dedicated to work leading possibly to the reported cases in the literature of increasing stress and burnout during the pandemic. These effects were found to be similar to all academics, independently of their gender and disciplinary field. More concerning is that this increased number of hours worked per week appears to have established itself as part of the new normal. The main driver for the increased workload is associated with teaching, and to a lesser extent with administrative duties.

## Introduction

The Covid-19 pandemic became an unprecedented global shock that affected campuses from all kinds of higher education institutions around the world [1]. The pandemic shock arrived as a crisis and brought new challenges and significantly increased the level of uncertainty for universities and academics [2]. In 2020, many universities were forced to significantly limit or even stop campus activities, with these activities still significantly limited in 2021. Conceptually, the Covid-19 pandemic assumed the characteristics of a total social fact as defined by Mauss [3], that is, an event that had an immediate, almost simultaneous, and direct impact on the professional, economic, familial, and social dimensions of everybody’s lives. In this sense, one can argue that this pandemic is a unique crisis, but also one that because of its total social fact characteristics is an interesting one to understand the effects of crises on academic work.

A fast-growing stream of literature addresses the perceived effects of the COVID-19 pandemic on academics’ work and life (e.g., [4–7]). Most academics’ research came to a temporary standstill, and research projects—whether funded or not—had to be rescheduled. Academics adapted by moving from specific research phases to other stages, to writing papers based on analyses of the available data, or to writing grant proposals rather than collecting new data [6, 7]. Teaching also shifted online. Although distance learning and online education have existed for several decades and are relatively standardized teaching modes [8], many academics lacked any relevant experience and generally felt unprepared to deal with them. Several authors [4, 9–11] have highlighted the heightened uncertainty that this brought to teaching duties, adding that many academics were at a loss for how to organize the learning process, what platform to choose, and how to evaluate students and provide them with valuable feedback. Academics’ involvement in management and services also changed. The number of online meetings increased in most organizations, including universities [12], due to the impossibility of holding in-person gatherings. This has since led to “videoconference fatigue” [13], which has reportedly severely disrupted people’s work–life balance, and as such has also negatively affected other facets of work.

The findings from the studies above overwhelmingly underline how academics and/or their universities strategized to handle the crisis. They underscore the strain that was placed on academic work, the transfer of learning activities from in-person to virtual spaces and interactions, the conflicting and often difficult work–life balances that highlighted existing inequalities, and situations of stress and burnout [2, 14–16]. Most of these studies focused on teaching as the educational activity that was most affected and that required most of the adaptations. The situation has prompted much useful research into strategies for teaching using information technology [17]. Stress and strains resulting from adapted working modes and greater number of hours were identified in both teaching and research activities, although the former is usually considered to have demanded more of academics [18]. There were also perceptions that women academics may have endured a more complicated life due to the pandemic, or that the pandemic may have affected academics in some academic fields than others [15].

While the studies have merit, they tend to be mostly descriptive, relatively space-time limited, and rooted in personal experiences and expectations concerning the present and the future. This study complements these studies, by implementing a temporal quasi-experimental approach, where academics from all areas of knowledge and working around the world, were randomly selected and contacted in two points in time during 2020 (4 months apart) and asked to respond to a time-occupation survey. The survey was designed to respond to the two research questions of this study: 1) In what way, has the Covid-19 crisis affected the workload and task-time allocation of this work for academics? 2) Were some academics more affected in this regard than others? In order to obtain data to inform the analysis on these questions, participant academics were asked to report concerning a specific academic task, say for example, supervisory work, how much time they would have expected to spend on this activity before the pandemic (as if the pandemic had never existed) and what time they were currently spending on it. Although this time allocation is self-reported (drawing on the respondents’ perceptions), it allows for a more objective and broader assessment of influences that the pandemic crisis might have had concerning academic work and focus.

## Method

### Sample

The sample results from a randomly selected population of academics that are active in research. The process to collect these data was as follows. First, we obtained the names of all the authors who published a document that was indexed by Scopus in 2019. These documents include articles in peer-reviewed journals, conference papers, reviews, book chapters, notes, letters, and other types of documents. This assured not only that the randomly selected academics would be from a large variety of countries worldwide, but also from a wide spectrum of disciplinary fields. The choice for Scopus as the data source for our data relates to the fact that it is an indexing database that has a good coverage of the most reputable journals in all fields of knowledge [19]. The search for authors in 2019 relates to the fact that we wanted academics that were recently active in research at the time of the survey. The search warranted almost three million documents, from which one hundred and twenty-six thousand academics were randomly chosen and then sent a questionnaire. The corresponding author and his or her e-mail of every three documents published was selected, in a process that continued until the names and e-mails of one hundred and twenty-six thousand academics was reached. Since Scopus does not structure the documents per disciplinary field (unless one requests it), the randomness per disciplinary field was also assured. The total number of one hundred and twenty-six thousand was chosen because the authors were expecting a very low response rate considering that: 1) response rates to online surveys have been consistently declining [20], 2) the target population was enduring a period of uncertainty and change, which made them less willing to respond to questionnaires [21], and 3) the fact that the study had a longitudinal perspective and it was necessary to aim for a large initial number of potential respondents as possible, because substantial non-response rates in the second wave of the questionnaire were to be expected, as indicated by the literature [22]. Of the one hundred and twenty-six thousand e-mail invitations sent via an online survey platform, 14% were returned as undeliverable. All the participants provided written consent to participate in the study, by ticking a box in the first page of the online survey where the conditions of participation and goals of the research were explained. Participation was voluntary and the participants could withdraw at any time without any consequence. All the participants of the study are academics. There was a filter in the questionnaires in order to keep only those who worked at the university.

The questionnaire was designed by the authors of the study, and contained questions related mostly to workload, work allocation in the current and previous semester, and expected and actual allocation of time allocated on a variety of research, teaching and management/service tasks. A few questions were focused on the characteristics of the academics, such as gender, disciplinary field and country where they were working. The questionnaire was as concise as possible to try and maximize complete responses and directed to focus on questions that were key to provide critical information to this study. The questionnaire was first implemented in May, and then again in November in 2020, each time in a single wave.

In the first wave of the survey, 902 participants completed the questionnaire. However, handling missing data through listwise deletion reduced the effective working sample to 525 participants. The most heavily represented countries were the United States of America (N = 104; 19.8%), Brazil (N = 49; 9.3%), and Italy (N = 31; 5.9%), with the remaining participants distributed over a series of other countries. On average, the participants reported having held a Ph.D. for 18 years. They reported working a median of 47 hours per week in pre-pandemic semesters and a median of 51 hours per week in the first semester during the pandemic. In the second wave of the survey, a total of 417 participants completed the questionnaire. Again due to missing data, the effective working sample was smaller and consisted of 169 participants. The most heavily represented countries in this wave were once again the U.S. (N = 42; 24.9%), Brazil (N = 16; 9.5%), and Italy (N = 11; 6.5%). The average time since the respondents’ Ph.D.s were awarded was still 18 years. They reported working a median of 51 hours per week in the first semester during the pandemic, and 46 hours in the second semester of the pandemic. The demographic characteristics for both waves, in terms of field of science and gender, are shown in Table 1.

**Table 1:** Sample characteristics by field of science and gender.

	Wave 1		Wave 2	
	N	%	N	%
Field of Science				
Natural and Mathematical Sciences	88	16.8%	25	14.8%
Health Sciences	125	23.8%	42	24.9%
Engineering & Technology	75	14.3%	18	10.7%
Social Sciences	197	37.5%	71	42.0%
Humanities and Arts	40	7.6%	13	7.7%
Gender				
Male	311	59.2%	101	59.8%
Female	214	40.8%	68	40.2%

### Time frames

Although the data was collected in two waves, the survey encompasses three time periods. In the first wave, participants were asked how many hours per week they spent on several activities on the last semester (pre-pandemic period). The pre-pandemic semester will be referred to as t_0_. Additionally, in the first wave participants were also asked how many hours per week they spent on several activities, and how many would they spend had this been a regular semester. This semester, the first in the pandemic period, will be referred to as t_1_. Finally, in the second wave, participants were asked the same questions regarding the ongoing semester–the second in the pandemic period–which will be referred to as t_2_.

### Data processing

To ascertain whether semestral changes were due to the pandemic effect, or simply usual changes, two different metrics were created and subsequently used for pairwise-comparisons–Δ_*obs*_ and Δ_*exp*_. The first is the change in working hours observed from a given semester, relative to the last semester. For example, for any given activity, the Δ_*obs*_ for the first pandemic semester and the pre-pandemic period is given by:

$$\Delta_{obs}=x_{t_{1}}-x_{t_{0}}$$ (1)

Where x is the estimated number of weekly working hours for that activity. Similarly, the Δ_*exp*_ for that time period comparison is given by:

$$\Delta_{exp}=y_{t_{1}}-x_{t_{0}}$$ (2)

Where y is the estimated number of weekly hours in a normal situation, and x is the estimated number of weekly working hours. This structure allows comparison between what would be the typical working hour changes, and the real working hours changes, permitting isolation of the pandemic effect on working hours inflation or deflation.

For comparisons between the second and the first pandemic semester, the formula for Δ_*obs*_ is identical, changing merely in the time frame:

$$\Delta_{obs}=x_{t_{2}}-x_{t_{1}}$$ (3)

However, Δ_*exp*_ changes in one of its parameters, in that it does not rely on the previous semester’s observed working hours, but rather on the expected working hours if it had been a regular semester. This is because Δ_*exp*_ aims to provide a baseline scenario of how the changes would have been like in a non-pandemic period, and while for t_0_ the effect hours were available, for t_1_ it is necessary to use the expected working hours in order to establish a continuum of estimated non-pandemic changes. As such, the formula for Δ_*exp*_ in these time frames comparisons is:

$$\Delta_{exp}=y_{t_{2}}-y_{t_{1}}$$ (4)

**Fig 1:**
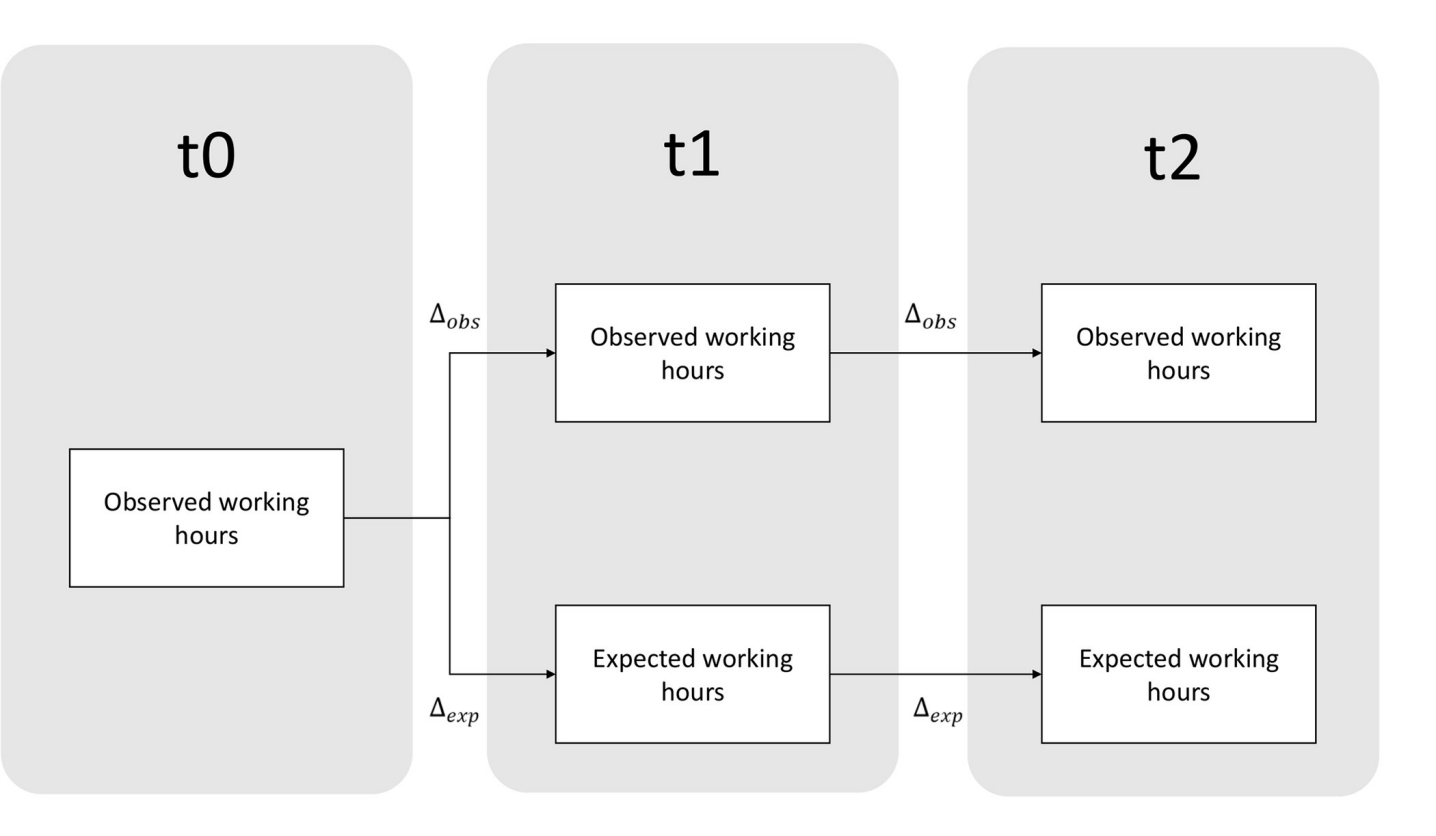
Diagram of time periods and changes calculations.

Where the notation is identical to what is noted above. Fig 1 aims to illustrate how the changes were computed in reference to the various time periods:

### Procedure

We employed a repeated measurements general linear model [23] in order to evaluate differences between Δ_*obs*_ and Δ_*exp*_. This is a within-subjects factor which was noted in the model as “differences”. The model included Gender, Field of Science, and Country as between-subjects fixed factors for control purposes. For each activity, estimated marginal means were produced. For descriptive purposes, a new variable was subsequently created for each activity—Δ_*diff*_–which is the difference between the estimated marginal mean for Δ_*obs*_ and Δ_*exp*_–which represents the effective pandemic inflation / deflation in working hours after considering controls and the baseline expected changes. Two models will be described–one which compares the pre-pandemic semester with the first pandemic semester, and another which compares the first pandemic semester with the second pandemic semester.

### Limitations

The methodology used in this article brings several benefits. First, it allows us to estimate the pandemic inflation in working hours more accurately than simply comparing the various semesters without a baseline–as that would not tell us if those differences would have occurred anyway in a typical year. However, this strength is also, simultaneously, the major weakness in this approach; since there is not any unafflicted countries in the world which could be used as a control group (as in a traditional experimental design), the only recourse was inquiring participants on what would have happened normally. In other words, what serves as a control group is the participant’s own “what-if” scenarios. This, of course, incurs the potential of introducing bias since the participants are providing an educated estimate, but not necessarily the effective documented working hours. Unfortunately, this is a limitation which needs to be acknowledged but also one that cannot realistically be addressed–the pandemic has impacted every country in the world, and as such there is no way to obtain a “true” control group in the traditional sense. Another limitation that needs to be considered is that aspects of the family, work-life balance, and other equally important dimensions are not considered. Although some of these variables were included in the survey, they were excluded from the analysis as our sample size, especially for the second comparison, would not realistically be able to accommodate more variables. Nevertheless, we tested other variables independently, and found no significant effects or interactions that would justify their inclusion in the model. As such, the only controls we employed are the three more common ones: gender, country, and field of science.

Furthermore, it must be noted that, despite our best efforts, the response rate was quite low. This may raise issues regarding the representativeness of the sample and the generalizability of the findings. Although this should be taken into consideration, we note that the characteristics of our sample of academics and scientists, at least in terms of gender and field of science, are similar to those of other papers with larger samples (e.g., [24, 25]). Although this does not necessarily mean that sampling bias is absent, it should at least mitigate some of the concerns about the representativeness of the sample.

## Results

### Pre-pandemic period versus first pandemic semester

Multivariate statistics for the within-subjects factors were verified prior to analysis of the univariate comparisons. Differences between observed and expected changes were noted as highly significant (Pillai’s Trace = 0.147, F(13, 475) = 6.296, p < 0.001), indicating that at least one of the activities exhibited significant univariate effects. Gender exhibited no significant interaction with the differences (Pillai’s Trace = 0.030, F(13, 475) = 1.135, p = 0.326), indicating that these are consistent for both men and women, which is a similar finding to the disciplinary fields interaction, also non-significant (Pillai’s Trace = 0.107, F(52, 1912) = 1.012, p = 0.451). Country did exhibit a significant interaction (Pillai’s Trace = 1.041, F(416, 6331) = 1.325, p < 0.001), but due to the significant amount of participating countries such a type of national level comparison would be beyond the scope of the current paper, and as such the variable is merely noted for its contribution to the analysis as a control. The results for the multivariate analysis are summarized in Table 2.

**Table 2:** Multivariate tests for the pre-pandemic versus first pandemic semester comparison.

	Pillai’s T	F	Df	Error df	p-value
Between-subjects effects					
Gender	0.010	3.833	13	475	0.975
Country	0.889	1.117	416	6331	0.055
FOS	0.096	0.902	52	1912	0.672
Within-subjects effects					
Time	0.147	6.296	13	475	<0.000
Time * Gender	0.030	1.135	13	475	0.326
Time * Country	1.041	1.325	416	6331	<0.000
Time * FOS	0.107	1.012	52	1912	0.451

**Fig 2:**
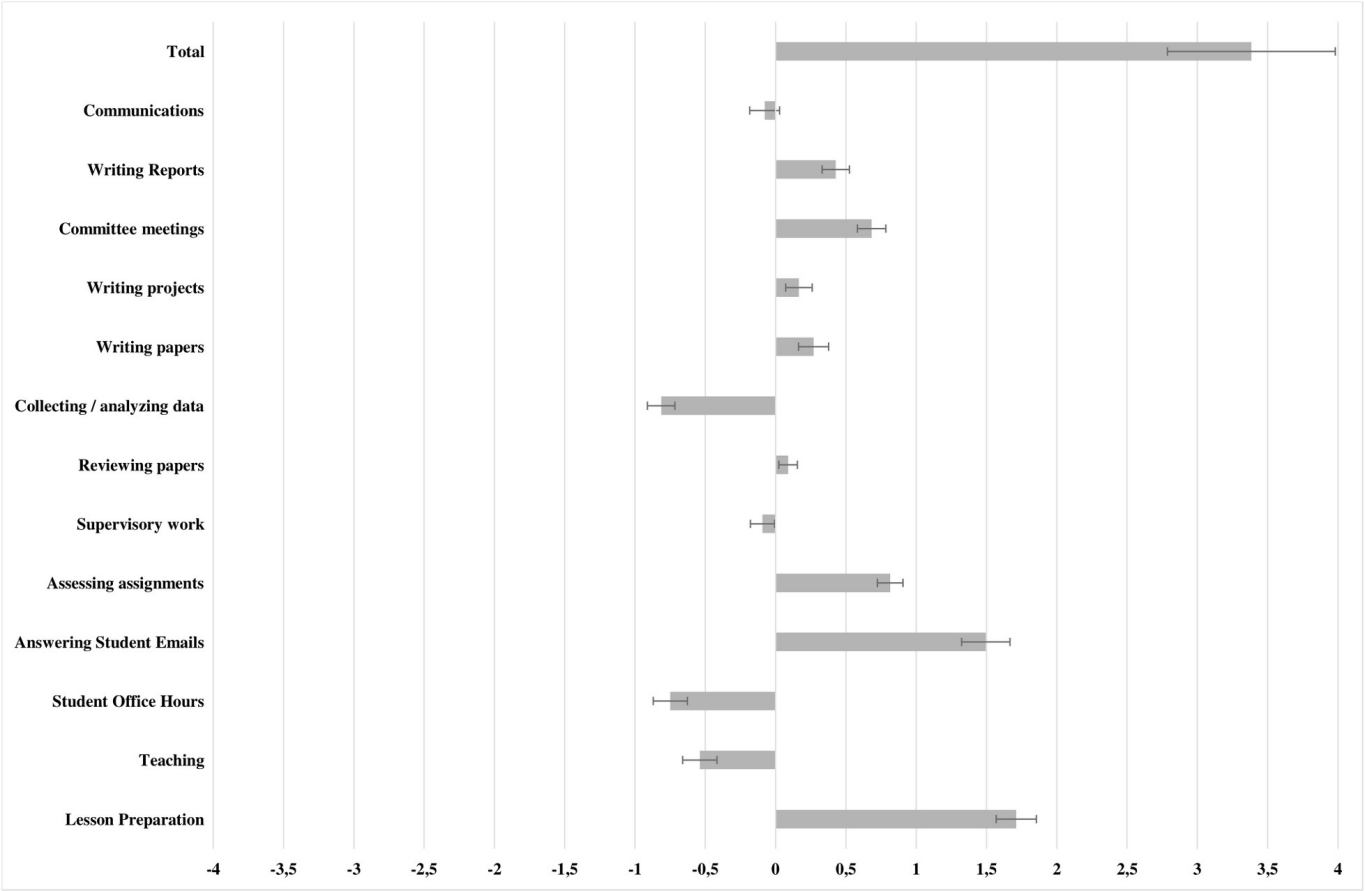
Differences between observed and expected changes in working hours per week in the first pandemic semester, relative to the previous semester. Error bars indicate 95% confidence intervals.

In this comparison, several important differences emerged, largely relating to teaching, which is expected due to the necessary adaptation of physical classwork to online formats. The number of hours per week dedicated to lesson preparation increased (Δ_diff_ = 1.712, F(1, 487) = 24.202, p < 0.001). Simultaneously, the number of hours dedicated to teaching slightly decreased (Δ_diff_ = -0.539, F(1, 487) = 5.462, p < 0.05), as did the number of student office hours (Δ_diff_ = -0.749, F(1, 487) = 11.518, p < 0.01). The latter is partly explained by the substantial increase in time dedicated to answering student emails, as student queries likely migrated to this format (Δ_diff_ = 1.495, F(1, 487) = 36.521, p < 0.001). The number of hours spent assessing assignments also increased (Δ_diff_ = 0.815, F(1, 487) = 23.509, p < 0.001). Finally, the only non-teaching aspect which evidenced significant changes is the number of weekly hours spent on committee meetings, which increased as well (Δ_diff_ = 0.683, F(1, 487) = 8.560, p < 0.01). In a more global comparison, average weekly working have increased by 3.384 across semesters (F(1, 487) = 10.878, p < 0.01) after accounting for controls and expected changes. These results are summarized in Table 3, whereas Fig 2 illustrates the differences between the observed and expected working hours changes relative to the previous semester.

**Table 3:** Comparison of observed and expected changes in working hours, comparing the first wave time period to the pre-pandemic period.

	Δ_obs_		Δ_exp_				Δ_diff_	
	M	SD	M	SD	F	p	M	SD
**Lesson Preparation**	**1.377**	**1.728**	**-0.335**	**1.094**	**24.202**	**0.000**	**1.712**	**1.668**
**Teaching**	**-0.881**	**1.794**	**-0.341**	**1.039**	**5.462**	**0.020**	**-0.539**	**1.417**
**Student Office Hours**	**-0.773**	**1.420**	**-0.024**	**0.424**	**11.528**	**0.001**	**-0.749**	**1.419**
**Answering Student Emails**	**1.431**	**1.825**	**-0.064**	**0.559**	**36.521**	**0.000**	**1.495**	**2.010**
**Assessing assignments**	**0.903**	**1.313**	**0.088**	**1.136**	**23.509**	**0.000**	**0.815**	**1.059**
Supervisory work	0.206	1.085	0.300	0.510	0.985	0.322	-0.093	0.999
Reviewing papers	0.055	0.909	-0.033	0.622	1.776	0.183	0.089	0.760
Collecting / analyzing data	-0.608	1.214	0.207	0.651	2.547	0.111	-0.814	1.143
Writing papers	0.535	1.470	0.266	1.053	0.012	0.914	0.270	1.239
Writing projects	0.162	1.101	-0.004	0.665	0.623	0.430	0.166	1.096
**Committee meetings**	**0.682**	**1.041**	**-0.002**	**0.570**	**8.560**	**0.004**	**0.683**	**1.181**
Writing Reports	0.400	1.299	-0.029	0.547	1.164	0.281	0.428	1.137
Communications	0.065	1.186	0.144	0.391	1.480	0.224	-0.078	1.234
**Total**	**3.560**	**7.658**	**0.171**	**3.384**	**10.878**	**0.001**	**3.384**	**6.962**

Notes: A repeated measures general linear model is shown. Estimated marginal means are shown, after controlling for Gender, Country, and Field of Science. Significant effects highlighted in bold.

### First pandemic period versus second pandemic period

As before, multivariate statistics for the within-subjects factors were verified prior to analysis of the univariate comparisons. When contrasting the first with the second pandemic semesters, substantially different results are observed. Notably, the difference between this and the previous semester in terms of working hours ceases to be different from the expected differences in normal times, as evidenced the lack of significant effects at a multivariate level (Pillai’s T = 0.102, F(13, 120) = 1.052, p = 0.407). At an univariate level, the notable exception is a very modest decrease in time spent reviewing papers (Δ_diff_ = -0.086, F(1, 132) = 5.124, p < 0.05). Gender maintains its non-significant interaction with the semestral differences (Pillai’s T = 0.108, F(13, 120) = 1.120, p = 0.349), as does the disciplinary fields (Pillai’s T = 0.346, F(52, 492) = 0.896, p = 0.679), indicating that these effects are consistent across genders and disciplinary fields. Country, as before, reveals a significant interaction (Pillai’s T = 2.978, F(403, 1716) = 2.978, p < 0.01). The results for the multivariate tests are shown in Table 4.

**Table 4:** Multivariate tests for the first and second pandemic semesters comparison.

	Pillai’s T	F	df	Error df	p-value
Between-subjects effects					
Gender	0.036	0.350	13	120	0.982
Country	3.004	1.280	403	1716	0.001
FOS	0.253	0.638	52	492	0.977
Within-subjects effects					
Time	0.102	1.052	13	120	0.407
Time * Gender	0.108	1.120	13	120	0.349
Time * Country	2.978	1.265	403	1716	0.001
Time * FOS	0.346	0.896	52	492	0.679

**Fig 3:**
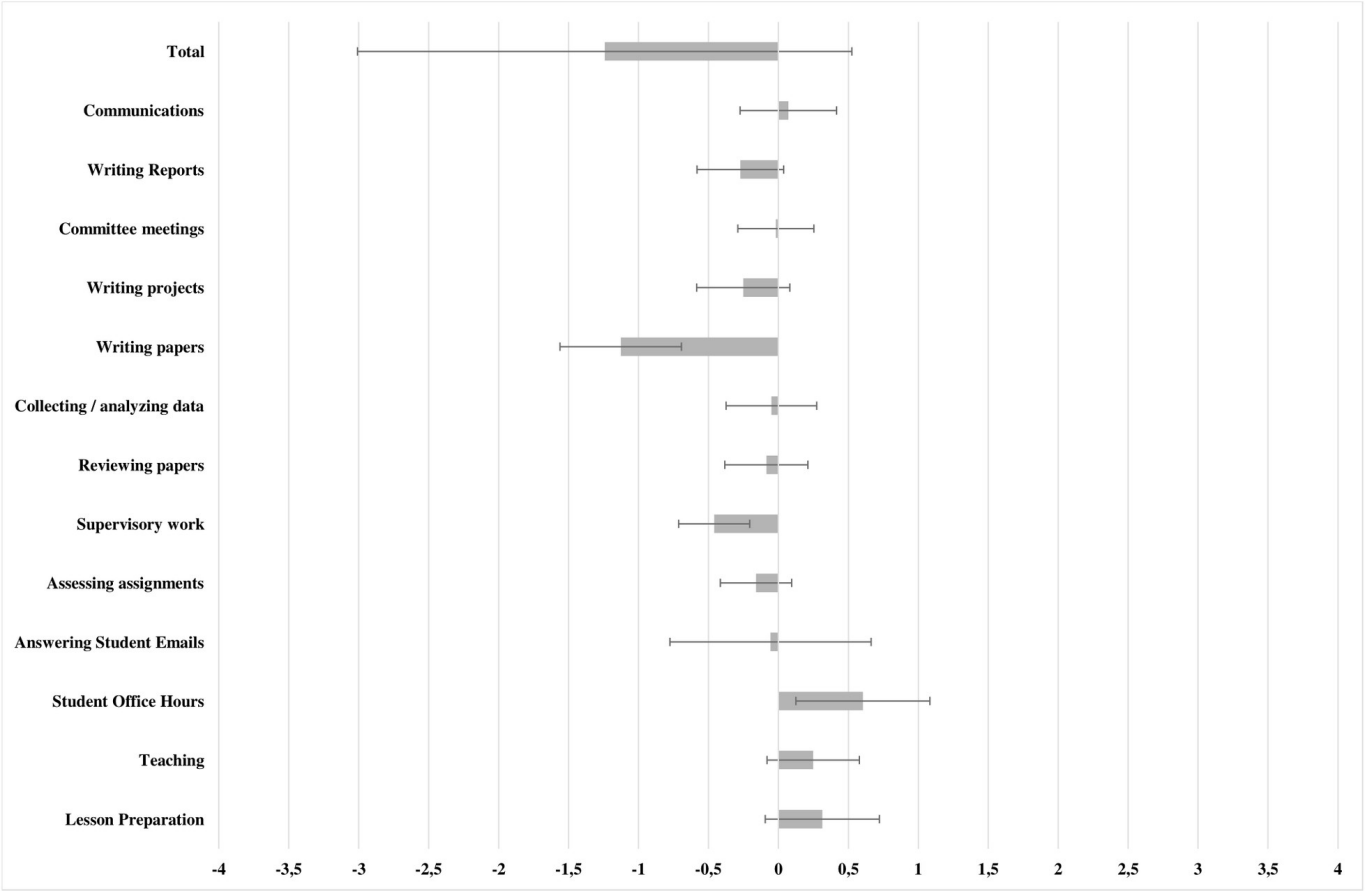
Differences between observed and expected changes in working hours in the second pandemic semester, relative to the previous semester. Error bars indicate 95% confidence intervals.

These findings do not mean that intensity of activity has fallen back to pre-pandemic levels, since the changes are calculated in reference to the previous time period. In fact, it represents the opposite–after a surge working hours were attained in the first pandemic semester, they appear to have been maintained in the second semester, since the observed differences between the second and the first pandemic semesters were similar to what would have occurred in a typical transition between the first and second semester–in other words, following the initial surge in working hours moving from the pre-pandemic period to the first semester, the working hours changed as usual but with reference to a new, higher, baseline level. In the global assessment, no differences were noted regarding the previous semester in terms of working hours after accounting for controls and expected changes (Δ_diff_ = -1.242, F(1, 132) = 0.003, p = 0.954). Table 5 shows the model for this analysis, while Fig 3 illustrates the differences between the observed and expected working hours changes relative to the previous semester.

**Table 5:** Comparison of observed and expected changes in working hours, the first and second pandemic semesters.

	Δ_obs_		Δ_exp_				Δ_diff_	
	M	SD	M	SD	F	p	M	SD
Lesson Preparation	-0.904	5.860	-1.217	5.708	0.021	0.885	0.314	2.682
Teaching	0.226	5.474	-0.022	5.987	2.058	0.154	0.249	2.169
Student Office Hours	0.469	1.893	-0.135	3.525	2.040	0.156	0.604	3.154
Answering Student Emails	-0.695	4.681	-0.639	3.983	0.740	0.391	-0.056	4.734
Assessing assignments	-0.799	4.818	-0.639	4.745	0.623	0.431	-0.160	1.683
Supervisory work	-1.235	4.881	-0.777	4.885	1.410	0.237	-0.459	1.674
**Reviewing papers**	**-0.670**	**4.691**	**-0.584**	**3.078**	**5.124**	**0.025**	**-0.086**	**1.955**
Collecting / analyzing data	-0.251	4.934	-0.201	4.773	0.038	0.846	-0.050	2.134
Writing papers	-1.447	4.648	-0.320	4.994	0.025	0.874	-1.127	2.855
Writing projects	-1.225	4.503	-0.974	4.871	0.028	0.867	-0.251	2.186
Committee meetings	-1.075	3.594	-1.057	3.406	0.000	0.994	-0.018	1.796
Writing Reports	-0.857	2.829	-0.586	2.792	0.850	0.358	-0.272	2.046
Communications	-0.293	3.798	-0.364	4.056	1.116	0.293	0.071	2.269
Total	-8.757	47.066	-7.515	46.376	0.003	0.954	-1.242	11.636

Notes: A repeated measures general linear model is shown. Estimated marginal means are shown, after controlling for Gender, Country, and Field of Science. Significant effects highlighted in bold.

## Discussion and conclusions

In an abrupt situation of uncertainty, academics have readjusted their total working time as well as the time allocated to specific academic tasks. On average, academics worked three hours more per week in 2020 than they were working in 2019. Considering that a typical semester has around 14 to 16 weeks, this means 42 to 48 hours worked more per semester, which is substantial considering that the academics in our sample worked around 51 median hours per week during the pandemic. This is much above the typical 35 working hours a week, and well above to what the literature considers to be long working hours, i.e, 40 working hours a week [26]. However, the pandemic’s effect on academics’ workloads only seems to have accentuated an existing situation in which academics work longer hours than usual, and they do so in many countries despite the potential health risks [27]. The literature already underlines the long working hours that academics put in even before the pandemic [28], with the pandemic exacerbating a situation that has now become a persistent “new normal”.

The main driver explaining the increased number of hours relates to teaching. This was particularly evident in the first semester of 2020, when academics were required to quickly adapt their teaching methods from face-to-face to online learning, as well as to change the way they communicated with the students outside the “classroom”. This finding is aligned with the existing literature that underlines the adaptation to new teaching modes as the most challenging of the changes that academics had to endure during the pandemic [4, 9, 11, 29]. Time on teaching shifted from lesson preparation to teaching proper, possibly because the focus was on redoing the curricula and pedagogical approach from a face-to-face to an online setting, and on learning and being trained in using online platforms to be able to teach [17]. Time on teaching likely declined because this adaptation led some courses to be streamlined and therefore reduced in size, and some pedagogical methodologies such as flipped learning, where the students read and prepare themselves before the class permitted more interactive classes where the instructor would not need to introduce content during the first part of the class [30]. The transition to online teaching, also implied a change in the assessment and evaluation of student work, and the assessment of assignments–some of them newly introduced–required a more intense scrutiny of the academics, which is typical of a transaction costs when one makes changes. Moreover, academics were used to and had established evaluation and feedback routines on the previous assessments when none existed for the new assignments [18]. The contact with the students also changed, from meeting them in office hours–usually at a determined day and time of the week–to answering to student e-mails that likely arrived throughout the week and at any given time and increased the work pressure that academics were feeling.

Management and service was affected to some extent, with time spent on committee meetings increasing. This was likely related to the need to strategize about teaching and learning activities, but also about other academic issues. Likely many of these meetings were information relative meetings where academics would be asked to participate to be acquainted with or receive instructions concerning health safety measures, teaching formats, online learning platforms, among others. Throughout the pandemic, time dedicated to research tasks remained unchanged except for an almost negligible decrease of time allocated to reviewing papers in the second semester of 2020. Unlike the perception brought by some literature on the pandemic that research activities were negatively affected, this study, at least from a time allocation perspective found no impact of the pandemic on research activities. Also not aligned with some findings, no differing effects were found between male and female academics in terms of time allocation to academic tasks. This means that women academics had increased the number of professionally allocated hours similarly to male academics. The analysis does not show, and it may have been the case that women academics may also have had compounded these hours with further additional hours taking care of children and household duties when this may not have been the case for men; this may have happened in more patriarchal societies, which likely increased substantially the burden and stress of female academics compared to male academics [31]. No findings were also found by disciplinary field, evidencing that from this perspective as well, the workload and work time allocation changes were similar to all academics.

Much of the literature on the perceived impact on the Covid-19 pandemic in academia, focuses on the opportunity presented to change teaching delivery and the introduction of new pedagogies and ways of doing academic work. Many studies also focused on the pressures that academics endured during the crisis. This study provides a complementary view, that basically sees the pandemic as a disruptive total event that perhaps had a role in accelerating and bringing to the fore some of the characteristics already perceived concerning academic work. Academics seem to have chosen to increase their working hours during the crisis as an adaptative behavior to mitigate uncertainty in a time of crisis. This may have been their own choice or because they felt pressured to do so–not necessarily by their university but perhaps also inspired by the behaviors of their peers. The result is that the working hours per week increased when this professional group already works above average working hours. Academics work long hours and are increasingly vulnerable to situations of stress and burnout, and our analysis suggests that a new normality was established during the pandemic where academics are working even more hours. This merits attention and raises the question: to what extent is this situation sustainable and what are the long-term consequences of this, when in other productive sectors, decreasing number of working days and hours are being seriously considered to foster productivity, work quality, and quality of life.
